# Effect of autologous umbilical cord blood transfusion in the development of retinopathy of prematurity: randomized clinical trial – study protocol

**DOI:** 10.3389/fped.2023.1269797

**Published:** 2023-10-12

**Authors:** Laura Torrejon-Rodriguez, Alejandro Pinilla-Gonzalez, Inmaculada Lara Cantón, Abel Albiach-Delgado, Mari Merce Cascant-Vilaplana, María Cernada, Julia Kuligowski, Maria Pilar Solves Alcaina, Inés Gómez, Maximo Vento, Marta Aguar Carrascosa

**Affiliations:** ^1^ Department of Neonatology, Instituto de Investigación Sanitaria La Fe (IISLAFE), Valencia, Spain; ^2^ Neonatal Research Group, Hospital Universitario y Politécnico La Fe (HULAFE), Valencia, Spain; ^3^ Department of Hematology, Hospital Universitario y Politécnico La Fe (HULAFE), Valencia, Spain

**Keywords:** retinopathy of prematurity, transfusion, prematurity, umbilical cord blood, fetal hemoglobin

## Abstract

**Background:**

Currently, the treatment of anemia in preterm infants is based on packed red blood cell (RBC) transfusions from adult donors. Oxygen (O2) is mainly transported to the tissues bound to hemoglobin (Hb). In extremely low gestational age neonates (ELGANs), fetal hemoglobin (HbF), which has a higher affinity for O2, represents up to 95% of circulating hemoglobin. During the first month of life, the majority of ELGANs will require an adult-donor RBC transfusion causing HbF levels to rapidly drop. HbA releases 50% more oxygen in peripheral tissues than HbF. Increased release of O2 in the retina is one of the main factors related to the development of retinopathy of prematurity (ROP). Collecting umbilical cord blood and using autologous umbilical cord whole blood (UCB) transfusions would contribute to maintaining physiological HbF concentrations in newborns and avoid oxygen-in-excess derived damage.

**Methods:**

This is a randomized, double-blinded, multicenter clinical trial. ELGANs ≤28 weeks of gestational age will be randomized 1:1 to receive an autologous umbilical cord blood transfusion (intervention arm) or standard transfusion of packed RBC from an adult donor (control arm) to assess ROP development. Assuming a 50% reduction in ROP incidence, 134 patients (67 per group) will be recruited. When blood transfusion is indicated, the Blook Bank will supply UCB or RCB according to the patient's group. The primary endpoint is the incidence of any ROP. Secondary endpoints are assessessment of treatment safety, results of biomarkers related to ROP and its chronology, and urine oxidative stress markers. In addition, the cellular composition of umbilical cord blood and its relationship with prematurity-related pathologies will be analyzed. All patients will be followed-up to 24 months of corrected age to evaluate their neurodevelopment.

**Discussion:**

ROP is a major cause of irreversible blindness in preterm newborns. Transfusions with adult donor blood can lead to complications, including ROP. UCB transfusions offer advantages by maintaining physiological HbF levels and potentially optimizing postnatal development. Moreover, autologous UCB transfusion could reduce risks associated with heterologous blood products, although volume collection remains challenging. UCB contains growth factors and progenitor cells that may impact ROP.

## Introduction

Retinopathy of prematurity (ROP) is a common comorbidity associated with extreme prematurity that affects up to 64% of extremely low gestational age neonates (ELGANs) below 28 weeks of gestational age (GA) (1, 2). ROP is a condition exclusive to preterm newborns and is the leading cause of blindness in ELGANs with significant neurodevelopmental implications in the short and long term (3).

Physiologic retinal development requires a relative hypoxemic microenvironment during intrauterine growth, where the arterial partial pressure of oxygen (PaO2) ranges from 30 to 40 mmHg. However, PaO2 rapidly increases to 100 mmHg whithin minutes after premature birth, hindering retinal blood vessel growth. In the first stage of ROP, hyperoxia suppresses the production of vascular endothelial growth factor (VEGF) and erythropoietin (EPO), leading to retinal vasoconstriction and localized hypoxia, ultimately causing delayed retinal development. Additionally, preterm birth causes the depletion of factors such as insulin-like growth factor 1 (IGF-1), which seems to contribute to the alteration of retinal vascular development (4, 5). In the second stage, the prolonged retinal hypoxia triggers the production of proangiogenic factors, leading to pathological and aberrant neovascularization that leads to the formation of fibrous scars and may potentially result in retinal detachment and subsequent risk of blindness or severe loss of visual acuity.

The severity of ROP inversely correlates with the degree of prematurity (6). Prematurity creates a favorable environment for this condition by exposing the immature retina to high oxygen levels. Despite extensive research conducted over the past six decades to investigate the relationship between prematurity, oxygen, and ROP, the treatment strategies for ROP are focused on applying therapies once the pathology has been developed such as laser therapies or intravitreal injection of bevacizumab (7, 8). However, no effective strategy has been found to significantly prevent the progression of ROP before its onset. Limited supplemental oxygen has been evaluated as a potential intervention, but this practice has the counterpart of increasing mortality (9, 10).

Red blood cell (RBC) transfusions have been associated with a higher incidence of ROP, and its severity is directly related to the volume of blood transfused (11). In term newborns, fetal hemoglobin makes up about 60%–80% of total hemoglobin. In ELGANS, fetal hemoglobin makes up 90%–95% of total hemoglobin (12). Although the physio-pathological mechanism is unknown, it is postulated to be linked to the significant increase in adult hemoglobin (HbA) levels. HbA releases up to 50% more oxygen than fetal hemoglobin (HbF) to peripheral tissues. Because blood from bank donors comes exclusively from adults, RBC transfusions increase HbA levels generating a hyperoxic stimulus in the retinal capillaries that could trigger the development of ROP (13–15). Moreover, low HbF levels have also been associated with ROP (16, 17). Additionally, recent studies have suggested that HbF could protect against oxidative stress due to its biochemical properties (18, 19).

Based on these premises, the utilization of umbilical cord blood transfusion (UCB) from healthy newborns has been proposed to prevent ROP in preterm infants (6). This approach is feasible and safe, although it is not exempted from the inherent risks associated with allogeneic transfusions (20). As an alternative, autologous UCB transfusion is suggested for treating anemia in ELGANs. Autologous UCB transfusions (UCBa) have been widely employed in full-term infants with congenital heart diseases requiring extracorporeal surgery (21, 22). The safety of UCBa has been extensively demonstrated, with no adverse effects reported in numerous studies involving over a hundred transfused patients (23–26). Furthermore, the transfusion risks associated with using allogeneic blood are eliminated in this approach. In premature patients, less information is available. However, most studies have shown that a volume ranging from 20 to 25 ml/kg is usually collected, meeting the requirements for at least one to two autologous transfusions (24, 25). Not only has UCBa been seen as equivalent to adult-donor RBC in increasing hematocrit (24), but this approach could also sustain HbF levels during the critical period of extremely preterm infants’ development. Additionally, UCB is enriched with self-hematopoietic growth factors, hematopoietic stem cells, and mesenchymal stem cells, which play a crucial role in developing connective tissue and blood vessels (27). Some authors have hypothesized that these cells could modulate angiogenesis and promote an appropriate retinal microenvironment, potentially reducing the progression of ROP (27–29).

We hypothesize that using UCBa in the first weeks after birth will contribute to keeping elevated HbF blood concentrations and subsequently reduce the risks inherent to HbA of interfering with the growth of the retinal vascular system.

This study aims to assess the effect of UCBa transfusion vs. standard treatment with packed adult-donor RBC in the incidence of ROP. Secondary objectives are to determine the impact of UCB autologous transfusion on the expression of biochemical markers of ROP, to determine the effect of UCB autologous transfusion on oxidative stress, to characterize the specific composition of stem cells in UCB in preterm newborns and its relationship with complications of prematurity.

## Methods and analysis

### Study design

To test the hypothesis, we have designed a randomized, multicenter, double-blinded trial to evaluate the effect of UCBa transfusion on the development of ROP compared to transfusion of adult-donor RBC in preterm infants with GA ≤28 weeks. Patients will be randomized in a 1:1 ratio to receive either UCBa transfusion or standard treatment with adult-donor RBC.

**Figure 1:**
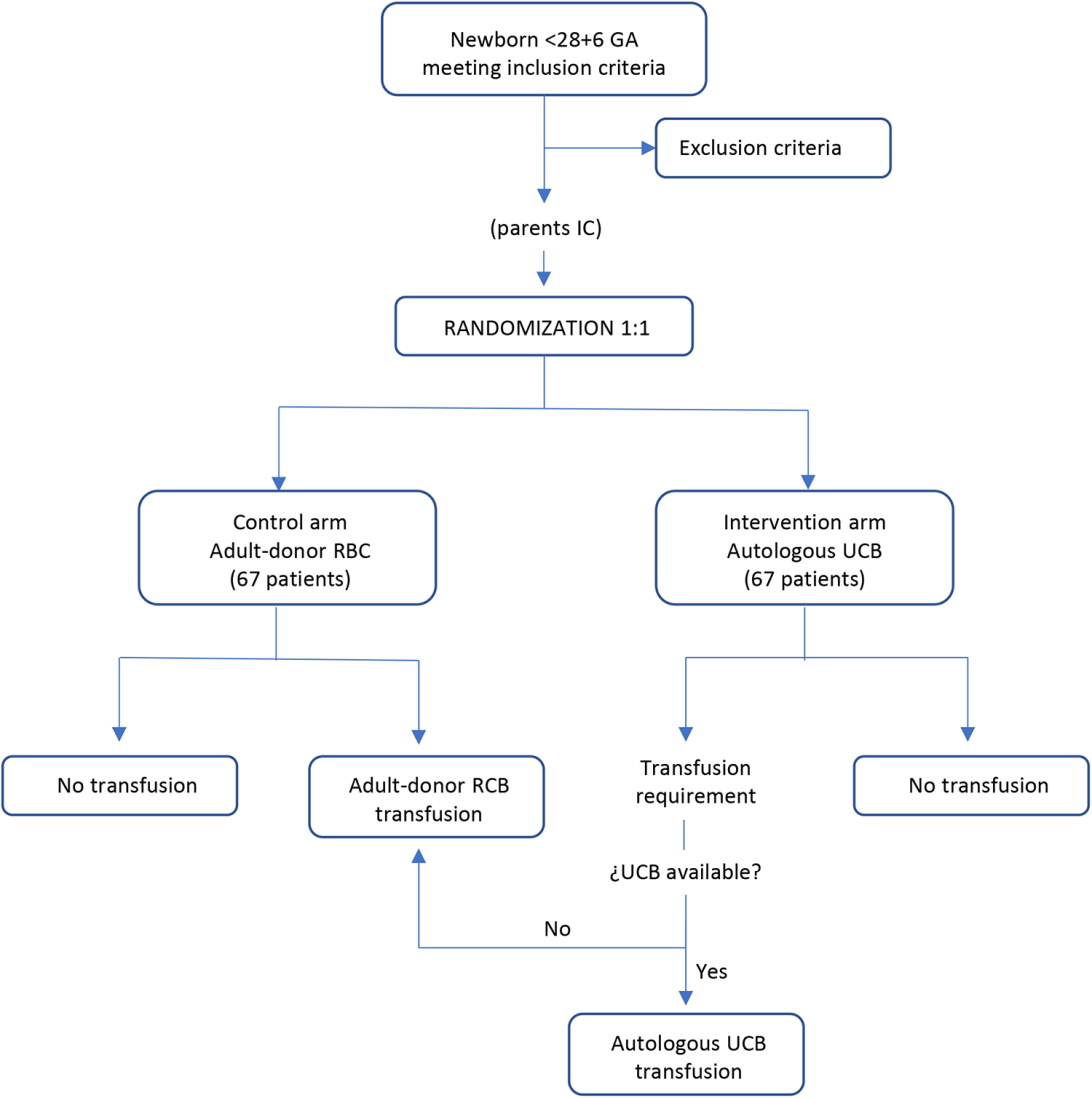
Flow chart illustrating intervention and control arm according to clinical trial protocol.

The study will be conducted at three hospitals: The University and Polytechnic Hospital La Fe (Valencia, Spain), the University Clinical Hospital of Santiago (Santiago, Spain), and the Maternal and Child University Hospital Complex of Las Palmas (Las Palmas, Spain). The protocol was written following the Standard Protocol Items: Recommendations for Interventional Trials (SPIRIT) guidelines. The study was registered at European Union Drug Regulating Authorities Clinical Trials Database (EudraCT) with the identifier number 2022-003821-22. Figure 1 shows the SPIRIT flow diagram of the study.

### Study population

Inclusion criteria: Preterm infants with GA ≤28 + 6 weeks of GA.

Exclusion criteria are one or more of the following: major congenital malformations, monochorionic twin pregnancy, chromosomal abnormalities, hemoglobinopathies, isoimmunization, critical condition at birth with a predicted fatal outcome, and refusal to participate and sign the informed consent.

Patients will be managed according to the usual protocols regarding oxygenation limits. The enrolled patients’ targeted oxygen saturation range will be 91%–95%. Transfusion thresholds will be carried out according to local guidelines.

### Intervention

Patients will be randomized to receive either whole UCBa, intervention group, or standard adult-donor packed RBC, control group. For patients with anemia requiring blood transfusion according to the Spanish Society of Neonatology's standards Committee threshold's recommendations (30), the responsible physician will make the corresponding request for transfusion indicating the volume needed. The Blook Bank will supply UCBa or RBC according to the patient's group, but bag labeling will be indistinguishable. In case there is not enough UCBa, RBC will be administered. As the maximum storage of UCB is 35 days, both groups will receive adult-donor RBC concentrates after this age. Transfusion's traceability will always be maintained through the e-Delphyn Transfusion Service software. UCB extraction and storage protocols are detailed in Appendix I.

### Primary outcome

The primary outcome is the incidence of ROP in both groups. Diagnosis and staging of ROP will be established according to “International Classification of the Retinopathy of Prematurity, Third Edition (ICROP3)”. ROP diagnosis will be made by an expert ophthalmologist using indirect ophthalmoscopy and RetCam imaging.

### Secondary outcomes

Outcomes related to effectiveness

- Recovery of hematocrit and hemoglobin values.
- Transfusion requirements during admission (number and volume of transfusions received).
- Treatment with EPO and other adjuvant treatments for anemia such as iron and folic acid supplementation (time of initiation, number of doses received).
- Assessment of post-transfusion tissue oxygen transfer status: Brain and somatic NIRS value, oxygen saturation measured by pulse oximetry, lactate, HbF levels.

Outcomes related to safety

- Incidence of acute transfusion reactions.
- Incidence of late complications: bronchopulmonary dysplasia, periventricular leukomalacia, necrotizing enterocolitis, hemodynamically significant patent ductus arteriosus, sepsis, and death.

Outcomes related to retinopathy markers

- Serum levels of EPO, VEGF and IGF-1.
- HbF levels.

Outcomes related to ROP treatment

- ROP severity
- Laser therapy
- Anti-VEGF administration

Outcomes related to UCB cellularity and oxidative stress.

- Quantification of reactive oxygen species in urine.
- Composition of stem cells in UCB.

Data concerning retinopathy and oxidative stress biomarkers quantification and stem cells typification is provided in Appendix II.

### Sample size calculation and power

We consider the development of ROP a competitive outcome with mortality due to its late onset during postnatal development. The combined variable death or ROP was considered the primary study variable. The incidence of death or ROP in our center in ≤28 weeks is 41%. A 50% reduction in the incidence will be considered clinically significant, so considering a significance level of 0.05 and a power of 0.8, the calculated sample size is 122 patients. Assuming a drop ratio of 10%, 134 patients will be recruited for the study (67 in each group).

University and Polytechnic Hospital La Fe is a tertiary center with 6,000–6,500 births annually. In addition, two other recruiting centers, University Clinical Hospital of Santiago (2,000–2,200 births annually) and Maternal and Child University Hospital Complex of Las Palmas (4,500–4,800 births annually), are included in the study, so we estimate that the sample size could be accomplished in the three years of the study duration.

### Recruitment and consent

Once a potential study participant has been identified, investigators will ask for informed consent from the infant's parents or legal guardians. Written informed consent will be obtained prenatally before delivery, reviewed, signed, and dated by the investigator and parents/legal guardians. The patient information sheet will include all the complementary tests carried out during the study.

### Randomization and blinding

Randomization will be done just before birth and generated by random numbers using the R® 3.5.1 software (R Development Core Team, Auckland, New Zealand). Patient numbers will be assigned sequentially by order of entry into the study. Randomization will be stratified into two groups by gestational age (GA) (23–25 and 26–28), using block randomization. The Biostatistics Unit will provide a randomization list for the corresponding Neonatology Service containing the patient number and another for the Blood Bank Service holding the related treatment branch and the patient number. The neonatologist in charge of the patient will assign the patient a number for the study, ensuring that it has not been given to any other patient. Once the number has been provided, it will be sent to the Blood Bank, responsible for keeping the randomization list containing all the information about it.

The Blood Bank will receive the UCB from the delivery room. It will verify that it meets the quality criteria established by the Blood Bank and aliquot it into one or several units of blood, depending on the volume available. Once a transfusion is prescribed for a participating patient, the Blood Bank will dispense either UCB or RBC according to the assigned group in the same container bag typically used in the Neonatology Unit to be indistinguishable from guaranteeing the blinding. The neonatologist will remain blind to the randomization group, as will the nursing staff and specialists in charge of patient care.

### Follow-up

The clinical evaluation will be performed during follow-up at 40 weeks, 12 months, and 24 months of corrected age. Weight, height, and head circumference will be measured, and the child's complete structured physical evaluation will be performed at each visit. A comprehensive neurodevelopmental assessment using the Bayley IV scale will be served at a two-year follow-up.

### Unblinding

The blinding will be opened in those emergency cases in which knowledge of the assigned treatment is essential for the medical care and well-being of the patient. In such a case, the researcher should note the date and reason for unblinding.

The researcher or neonatologist in charge will contact the Blood Bank Service or unblinded operator, and the patient's treatment information will be provided. The blinding will be opened once the last patient enrolled in the study has been discharged from the hospital so that the results can be statistically analyzed. The neurodevelopmental specialist performing the long-term follow-up will remain blinded until the 24-month corrected age control of the last patient included in the study.

### Data collection and study monitoring

A record must be completed for each patient included in the study. Data will be anonymized using a unique code for each participant. The critical document containing each patient's name related to the code number will be stored in each center's principal investigator's folder. Data will be kept in an institutional research location of the principal investigator, secured with a password or key for the period specified by legislation.

During the trial, periodic monitoring visits will be carried out by an external monitor independent from the research team to ensure that the protocol and good clinical practices are followed. The monitors can review the source documents to confirm that the data collected are accurate. The researcher and the institution guarantee the monitors direct access to the source documents and the relevant regulatory authorities for verification.

### Adverse events (AEs)

Considering the characteristics of the investigated product, adverse events related to transfusions are managed in compliance with the regulations specified by the National Transplant Organization. The initiation of the study has been reported to the National Transplant Organization.

### Statistical analysis

The following sets of analyses are planned: (1) All included patients, regardless of whether they were transfused or not; (2) All included patients who received transfusions, as per intention-to-treat (including intervention group patients who received adult donor blood transfusions due to unavailability of umbilical cord blood); (3) All included patients who received transfusions as per protocol (excluding intervention group patients who received adult donor blood transfusions). Secondary analyses will also examine the effect of the two treatment strategies on the described secondary variables. An interim analysis of the results will be conducted once half of the sample has been collected to assess the presence of any relevant safety or mortality outcomes. An external committee will be proposed to perform the calculations while maintaining blinding until evaluation.

Categorical variables are described with each category's numerical count (percentage). If the application assumptions are met, they are compared with Pearson's chi-square test, and if not, Fisher's exact test. Continuous variables are represented with Tuckey box plots. If the constant variables are standard (*p* > 0.05 in the Shapiro–Wilk test), they are described with the mean ± standard deviation. They are compared using Student's *t*-test, first testing the hypothesis of equality of variances using Levene's test. If abnormal, they are described with a median (p25, p75) and compared with the nonparametric Mann–Whitney *U*-test. Comparison between both groups of repeated measures over time is performed by a two-way analysis of variance (ANOVA) test, first testing Mauchly's sphericity hypothesis and rejecting it with *p* < 0.05. Survival times are expressed with their median [95% confidence interval (95% CI)], and between-group comparison is performed with the log-rank test. Throughout the study, a *p* < 0.05 is accepted as the limit of statistical significance. The magnitude of the effect is quantified with the risk difference (expressed as a percentage), and its precision is indicated with the 95% CI.

### Ethical considerations

All the procedures have been reviewed and approved by the IRB of the PI's hospital (Comité de Ética de Investigación con medicamentos; University and Polytechnic Hospital La Fe, Valencia, Spain), and the Approval Number is 2021-269-1 also by the local IRBs of the participating hospitals. The study will be conducted by the protocol, ICH guidelines, the applicable regulations and guidelines governing clinical studies in Spain, and the ethical principles from the Declaration of Helsinki.

## Discussion

Retinopathy of prematurity is an exclusive disease of preterm newborns and the leading cause of irreversible blindness in this group of patients. Due to the improvement in the survival of highly preterm newborns, this disease is expected to increase even more in the coming decades. Any intervention focused on reducing the incidence of this pathology will improve the quality of life of premature patients and significantly impact the direct and indirect costs that blindness entails in such early stages of life. Autologous cord blood transfusions represent a simple and cheap procedure that has already been evaluated in similar scenarios in the neonatal population. Its use is not only not expected to present side effects but also very likely to prevent the side effects associated with the compared intervention, which is of great interest.

Transfusions with packed red blood cells from adult donors are now unavoidable in the context of premature infants. Their administration has been associated with various complications, including ROP. The main potential advantage of umbilical cord blood is that it contains the same amount of HbF as newborns *in utero*. Thus, autologous UCB transfusions would maintain a physiological concentration of HbF during the first weeks of life, which has a greater affinity for oxygen and is more stable in an oxidative environment, maintaining similar oxygen transportation and tissue delivery as in the fetal stage. This fact could optimize the postnatal development of different immature tissues. Moreover, UCBa transfusion reduces the inherent risks of receiving heterologous blood products because the product is entirely compatible with the recipient.

Umbilical cord blood is rich in growth factors that play a unique role in the pathogenesis of this disease, as previously mentioned. It also contains progenitor cells that could promote better postnatal development. To confirm the influence of these factors, basic research studies and randomized clinical trials are needed. This trial will study all these factors and the expression of oxidative stress markers. Physiological mechanisms of retinopathy of prematurity will be analyzed, and a predictive model based on biomarker expression could be designed to apply targeted preventive and therapeutic strategies. Moreover, it will allow us to expand the insufficient knowledge regarding the cellular components of preterm cord blood and explore new lines of research.

The limitations of this study are inherent to a clinical trial and are mainly manifested in two aspects. Firstly, the difficulty of recruiting patients in the complex environment of Neonatal Intensive Care Units at the birth of a preterm patient, where uncertainty about potential complications of this condition and other concurrent factors makes it challenging to approach the parents of eligible patients. For this reason, a multicenter design has been implemented, including three clinical units with sufficient annual admissions. On the other hand, there might be cases where the volume of blood collected needs to meet the complete transfusion needs of the patient. It is assumed that the most significant benefit of autologous transfusion will occur within the first two weeks of life due to the pathophysiology of the disease, during which time transfusion needs will be met. However, allogeneic transfusions are usually required as a supplement to meet complete requirements. This hypothesis is reinforced by the results presented by Hellstrom et al. (18), who found that only the drop in HbF during the first week of life was related to ROP. Moreover, the statistical analysis is designed in analysis sets to assess the actual impact of this effect. On the other hand, improving the collection technique and experience could help improve these results. Our group is working on developing an optimized system for cord blood collection.

The relevance of the trial is based on the need to address ROP, a serious ocular complication that currently lacks an effective treatment. Clinical trials like the one presented can be the beginning of a paradigm shift in transfusion medicine, using neonatal blood for neonatal patients whose situation differs significantly from adult patients, thereby improving the quality of care and clinical outcomes in this patient population.

## Appendix I

Extraction of UCB: The instructed obstetrics or neonatology team will extract UCB at the moment of birth. It should be noted that late cord clamping is not contraindicated for this purpose.

UCB Storage: Autologous UCB will be stored in the Blood Bank Service following the usual protocol. It will be labeled and destined exclusively for autologous use. The UCB can be used for a maximum of 35 days. This UCB will be aliquoted according to the volume requested.

Quality control of the UCB: The determinations protocoled by the Blood Bank Service will be carried out on maternal and UCB samples. The decisions include the following:

- ABO and Rho blood group (D)
- Screening for irregular anti-erythrocyte antibodies by methods that detect clinically significant antibodies.
- Tests for the detection of infectious agents:
  ▪ Syphilis: serological tests.
  ▪ Hepatitis B: AgHBs, AcHBc, AcHBs and NAT.
  ▪ Hepatitis C: Anti-HCV and nucleic acid genomic amplification tests.
  ▪ HIV I/II: Anti-HIV I/II and NAT.
- Tests necessary to detect carriers of other infectious agents in certain donors due to their specific epidemiological circumstances.
- Blood culture.

## Appendix II

(1) Retinopathy biomarkers
  - VEGF: VEGF or VEGF-A will be determined weekly during the first six weeks of life. The blood will be collected and centrifuged to separate the serum. It will be stored at −80°C until processed with Human VEGF Quantikine ELISA Kit.
  - EPO: It will be determined at first, 21, and 35 days of life by using Immulite 2000 system.
  - IGF-1: Using the Liason system will determine it at first, 21, and 35 days of life.
(2) Oxidative stress biomarkers in urine
  Validated methods based on liquid chromatography coupled to tandem mass spectrometry (UPLC- MSMS) will be used. Integrated functions within the PLS Toolbox (Eigenvector Research), as well as in-house written parts, will be used for data modeling and comparisons between groups, using variable reduction methods based on principal component analysis (PCA) and classification methods, such as the analysis partial least squares discriminant (PLS-DA).
(3) Blood cell typing in umbilical cord blood
  To quantify the number of hematopoietic stem cells, mesenchymal and endothelial cells in cord blood samples will be collected in an EDTA tube. The analysis will be performed by flow cytometry (BD FACScanto II (Becton Dickinson) and Diva Software (Becton Dickinson Biosciences)”. Mesenchymal cells will be tested to ensure they meet the minimum definition criteria, including the culture of mesenchymal cells for this purpose.

## References

[1] Graziosi A Perrotta M Russo D Gasparroni G D'Egidio C Marinelli B Oxidative stress markers and the retinopathy of prematurity. J Clin Med. (2020) 9(9):2711. 10.3390/jcm9092711 32825796 PMC7563779

[2] Hong EH Shin YU Bae GH Choi YJ Ahn SJ Sobrin L Nationwide incidence and treatment pattern of retinopathy of prematurity in South Korea using the 2007-2018 national health insurance claims data. Sci Rep. (2021) 11(1):1451. 10.1038/s41598-021-80989-z 33446899 PMC7809441

[3] Ingvaldsen SH Hansen TI Håberg AK Moholdt V Evensen KAI Dammann O Visual function correlates with neurodevelopment in a population cohort of school-aged children born extremely preterm. Acta Paediatr. (2023) 112(4):753–61. 10.1111/apa.16667 36627478

[4] Hellström A Smith LE Dammann O. Retinopathy of prematurity. Lancet. (2013) 382(9902):1445–57. 10.1016/S0140-6736(13)60178-6 23782686 PMC4389630

[5] Chan-Ling T Gole GA Quinn GE Adamson SJ Darlow BA. Pathophysiology, screening, and treatment of ROP: a multi-disciplinary perspective. Prog Retin Eye Res. (2018) 62:77–119. 10.1016/j.preteyeres.2017.09.002 28958885

[6] Podraza W. A new approach to neonatal medical management that could transform the prevention of retinopathy of prematurity: theoretical considerations. Med Hypotheses. (2020) 137:109541. 10.1016/j.mehy.2019.109541 31901610

[7] Hartnett ME Penn JS. Mechanisms and management of retinopathy of prematurity. N Engl J Med. (2012) 367(26):2515–26. 10.1056/NEJMra1208129 23268666 PMC3695731

[8] Chow LC Wright KW Sola A, CSMC oxygen administration study group. Can changes in clinical practice decrease the incidence of severe retinopathy of prematurity in very low birth weight infants? Pediatrics. (2003) 111(2):339–45. 10.1542/peds.111.2.339 12563061

[9] Supplemental therapeutic oxygen for prethreshold retinopathy of prematurity (STOP-ROP), a randomized, controlled trial. I: primary outcomes. Pediatrics. 2000;105(2):295–310. 10.1542/peds.105.2.295 10654946

[10] Askie LM Henderson-Smart DJ Irwig L Simpson JM. Oxygen-saturation targets and outcomes in extremely preterm infants. N Engl J Med. (2003) 349(10):959–67. 10.1056/NEJMoa023080 12954744

[11] Hengartner T Adams M Pfister RE Snyers D McDougall J Waldvogel S Associations between red blood cell and platelet transfusions and retinopathy of prematurity. Neonatology. (2020) 117(5):1–7. 10.1159/000512020 33291117 PMC7845415

[12] Gavulic AE Dougherty D Li SH Carver AR Bermick JR Mychaliska GB Fetal hemoglobin levels in premature newborns. Should we reconsider transfusion of adult donor blood? J Pediatr Surg. (2021) 56(11):1944–8. 10.1016/j.jpedsurg.2021.04.018 34052004

[13] Rivera JC Dabouz R Noueihed B Omri S Tahiri H Chemtob S. Ischemic retinopathies: oxidative stress and inflammation. Oxid Med Cell Longev. (2017) 2017:3940241. 10.1155/2017/3940241 29410732 PMC5749295

[14] Stone WL Shah D Hollinger SM. Retinopathy of prematurity: an oxidative stress neonatal disease. Front Biosci (Landmark Ed). (2016) 21(1):165–77. 10.2741/4382 26709767

[15] De Halleux V Truttmann A Gagnon C Bard H. The effect of blood transfusion on the hemoglobin oxygen dissociation curve of very early preterm infants during the first week of life. Semin Perinatol. (2002) 26(6):411–5. 10.1053/sper.2002.37313 12537312

[16] Ratanasopa K Strader MB Alayash AI Bulow L. Dissection of the radical reactions linked to fetal hemoglobin reveals enhanced pseudoperoxidase activity. Front Physiol. (2015) 6:39. 10.3389/fphys.2015.00039 25750627 PMC4335259

[17] Tin W Milligan DW Pennefather P Hey E. Pulse oximetry, severe retinopathy, and outcome at one year in babies of less than 28 weeks gestation. Arch Dis Child Fetal Neonatal Ed. (2001) 84(2):F106–10. 10.1136/fn.84.2.f106 11207226 PMC1721225

[18] Stutchfield CJ Jain A Odd D Williams C Markham R. Foetal hemoglobin, blood transfusion, and retinopathy of prematurity in very preterm infants: a pilot prospective cohort study. Eye (Lond). (2017) 31(10):1451–5. 10.1038/eye.2017.76 28548651 PMC5639193

[19] Hellström W Martinsson T Morsing E Gränse L Ley D Hellström A. Low fraction of fetal haemoglobin is associated with retinopathy of prematurity in the very preterm infant. Br J Ophthalmol. (2022) 106(7):970–4. 10.1136/bjophthalmol-2020-318293 33547036 PMC9234406

[20] Bianchi M Giannantonio C Spartano S Fioretti M Landini A Molisso A Allogeneic umbilical cord blood red cell concentrates: an innovative blood product for transfusion therapy of preterm infants. Neonatology. (2015) 107(2):81–6. 10.1159/000368296 25401961

[21] Sarin K Chauhan S Bisoi AK Hazarika A Malhotra N Manek P. Use of autologous umbilical cord blood transfusion in neonates undergoing surgical correction of congenital cardiac defects: a pilot study. Ann Card Anaesth. (2018) 21(3):270–4. 10.4103/aca.ACA_194_17 30052213 PMC6078044

[22] Fernandez A Chasovskyi K. The use of umbilical cord blood for autologous transfusion in neonatal open heart surgery. J Cardiothorac Vasc Anesth. (2020) 34(2):483–8. 10.1053/j.jvca.2019.05.007 31151859

[23] Brune T Garritsen H Witteler R Schlake A Wüllenweber J Louwen F Autologous placental blood transfusion for the therapy of anaemic neonates. Biol Neonate. (2002) 81(4):236–43. 10.1159/000056754 12011567

[24] Khodabux CM von Lindern JS van Hilten JA Scherjon S Walther FJ Brand A. A clinical study on the feasibility of autologous cord blood transfusion for anemia of prematurity. Transfusion. (2008) 48(8):1634–43. 10.1111/j.1537-2995.2008.01747.x 18507748

[25] Jansen M Brand A von Lindern JS Scherjon S Walther FJ. Potential use of autologous umbilical cord blood red blood cells for early transfusion needs of premature infants. Transfusion. (2006) 46(6):1049–56. 10.1111/j.1537-2995.2006.00841.x 16734824

[26] Titkov KV. Autotransfusion of cord blood erythrocytes in newborns with malformations requiring early surgical intervention. Anesteziol Reanimatol. (2014) 59(6):38–43. 25831701

[27] Lee OK Kuo TK Chen WM Lee KD Hsieh SL Chen TH. Isolation of multipotent mesenchymal stem cells from umbilical cord blood. Blood. (2004) 103(5):1669–75. 10.1182/blood-2003-05-1670 14576065

[28] Safranow K Kotowski M Lewandowska J Machalińska A Dziedziejko V Czajka R Circulating endothelial progenitor cells in premature infants: is there an association with premature birth complications? J Perinat Med. (2012) 40(4):455–62. 10.1515/jpm-2011-0199 22752779

[29] Hansmann G Fernandez-Gonzalez A Aslam M Vitali SH Martin T Mitsialis SA Mesenchymal stem cell-mediated reversal of bronchopulmonary dysplasia and associated pulmonary hypertension. Pulm Circ. (2012) 2(2):170–81. 10.4103/2045-8932.97603 22837858 PMC3401871

[30] Boixa H Sánchez-Redondo MD Cernada M Espinosa Fernández MG González-Pacheco N Martín A Recommendations for transfusion of blood products in neonatology. An Pediatr (Engl Ed). (2022) 97(1):60.e1–8. 10.1016/j.anpede.2022.05.003 35725819

